# Clinical and genetic findings in autism spectrum disorders analyzed using exome sequencing

**DOI:** 10.3389/fpsyt.2025.1515793

**Published:** 2025-02-25

**Authors:** Ana Blázquez, Laia Rodriguez-Revenga, María I. Alvarez-Mora, Rosa Calvo

**Affiliations:** ^1^ Department of Child and Adolescent Psychiatry and Psychology, Institute of Neuroscience, Hospital Clínic de Barcelona, Barcelona, Spain; ^2^ Child and Adolescent Psychiatry and Psychology Research Group, Institut d'Investigacions Biomèdiques August Pi i Sunyer (IDIBAPS), Barcelona, Spain; ^3^ Department of Basic Clinal Practice, Pharmacology Unit, University of Barcelona, Barcelona, Spain; ^4^ Department of Biochemistry and Molecular Genetics, Hospital Clinic of Barcelona, Barcelona, Spain; ^5^ Translational Research Group in New Therapeutic and Diagnostic Strategies in Liver Diseases Institut d'Investigacions Biomèdiques August Pi i Sunyer (IDIBAPS), Barcelona, Spain; ^6^ Centro de Investigación Biomédica en Red de Enfermedades Raras (CIBERER), Instituto de Salud Carlos III, Madrid, Spain; ^7^ Department of Medicine, University of Barcelona, Barcelona, Spain; ^8^ Centro de Investigación Biomédica en Red de Salud Mental (CIBERSAM), Instituto de Salud Carlos III, Madrid, Spain

**Keywords:** Autism spectrum disorders, childhood, exome sequencing, dysmorphic features, hypotonia

## Abstract

Autism spectrum disorder (ASD) refers to a group of complex neurodevelopmental disorders and is characterized by impaired reciprocal social interaction and communication, as well as the presence of restricted interests and stereotyped and repetitive behaviors. As a complex neurodevelopmental disorder, the phenotype and severity of autism are extremely heterogeneous, with differences from one patient to another. Chromosome microarray (CMA) and fragile X syndrome analyses has been used as a powerful tool to identify new candidate genes for ASD. Methods: In the present study, CMA was first used to scan for genome-wide copy number variants in the patient, and no clinically significant copy number variants were found. Exome sequencing (ES) was used for further genetic testing. Results: ES was performed on 20 subjects. Eighty percent of our sample presented intellectual disability. Other co-occurring clinical conditions included speech disorders, psychomotor delay, the presence of dysmorphic features and medical co-morbidities. A pathogenic variant was identified in 10 patients (*ADNP*, *FBN1*, *WAC*, *ASXL3*, NR4A2, *ALX4, ANKRD1, POGZ, SHANK3* and *BPTF*). Patients with a positive finding in ES were more likely to present a dysmorphic trunk, more than three dysmorphic features, hypotonia, psychomotor delay and strabismus. Conclusions: ES offers expanded diagnostic options for patients with ASD who are negative on CMA. However, further studies are needed for a better understanding of ASD etiology and also the different phenotypes.

## 1 Introduction

Autism spectrum disorder (ASD) refers to a group of complex neurodevelopmental disorders and is characterized by different behavioral manifestations, including impairments in social communication, as well as the presence of restricted interests and stereotyped and repetitive behaviors (1).

Comorbid conditions such as multiple psychiatric disorders, anxiety, depression, attention deficit/hyperactivity disorder (ADHD), epilepsy, gastrointestinal symptoms/problems, sleep disorders, learning disability, obsessive compulsive disorder, intellectual disability (ID), sensory problems and other disorders have been found in ASD (2, 3).

The etiopathogenesis of ASD remains largely unknown but it is well recognized that ASD is a complex, highly heterogeneous disorder, both in clinical presentation and with reference to the complex risk architecture that involve the complex interaction of several genes and environmental risk factors. Rare inherited and *de novo* variants are major contributors to individual risk for ASD (4, 5).

Etiologic investigation begins with a careful medical, developmental-behavioral, and family history and a thorough physical and neurologic examination. The physical examination should include assessment of growth relative to typical curves (including head circumference), dysmorphic features, organomegaly, skin manifestations of neurocutaneous disorders, and neurologic abnormalities (6, 7).

Genetic evaluation should be recommended and offered to all families as part of the etiologic workup. In parallel with advances in genomic technology, the list of genes linked to autism is growing (8–10). Many of these genes are vital for communication between neurons and could be involved in controlling the expression of other genes (5). Available evidence suggests chromosomal microarray analysis (CMA) as a “gold standard” first‐level test in the genetic diagnosis of ASD. CMA identifies copy number variants (CNVs), which are DNA duplications or deletions that alter the function of genes, and offers a high diagnostic yield, ranging from 14–20%, for individuals with unexplained ID, ASD or multiple congenital anomalies. There is much evidence that CNVs detected by CMA are major contributors to ASD pathogenesis in up to 10%–15% of affected children (11, 12). Because fragile X syndrome (FXS) increases the risk for ASD, DNA testing for FXS should be recommended for all children with ASD, but especially for boys and children with a suggestive family history of male members with ID (7).

When the history and physical examination, CMA, and FXS analysis do not identify an etiology, the next step in the etiologic evaluation for ASD is exome sequencing (ES). ES has proven instrumental in providing a molecular diagnosis for many patients with a broad spectrum of previously undiagnosed genetic disorders in a cost- and time-effective way (11, 12). ES technology allows for the identification of single-nucleotide variants, including loss-of-function and missense pathogenic variants, which have been found to be associated with ASD in 8–20% of individuals (7).

However, despite the revolutionary advance of genetic testing, a high percentage of children and adolescents with ASD still lack a genetic diagnosis. The main aim of this study was to analyze differences found between ASD patients with pathogenic findings in ES and those without any significant finding in order to discuss which characteristics give the best performance for ES.

## 2 Methods

### 2.1 Subjects and procedure

Sample recruitment was carried out retrospectively, from 2019 to 2023, among patients under routine care in the Department of Child and Adolescent Psychiatry and Psychology (Institute of Neurosciences, Hospital Clinic, Barcelona). Patients with a previously abnormal karyotype in peripheral blood, a positive FXS study, or a confirmed suspicion of an identifiable syndromic entity were excluded from the study. As a first step, CMA was performed on the sample after discarding pathogenic CNVs.

Each individual had a genetic examination and clinical evaluation of their cognitive abilities and ASD symptoms. Dysmorphic features, behavioral phenotype and other signs were explored prior to performing genetic testing. The local IRB approved the use of their retrospective clinical and sociodemographic data.

### 2.2 Sequencing and bioinformatics analysis

Until 2021, ES libraries were prepared using the Illumina Nextera Flex for Enrichment assay (Illumina, San Diego, CA, USA), and subsequent paired-end sequencing (2 _ 150 bp) of the whole exome was performed with the Nextseq550 platform (Illumina).

From 2022, ES libraries were prepared using the DNA Prep with Enrichment (Illumina) and the SureSelect Human All Exon v8 kits (Agilent Technologies, Santa Clara, CA, USA). Paired-end sequencing (2 _ 150 bp) of the exome was performed using the Illumina NextSeq 2000 sequencer (Illumina, San Diego, CA, USA). Briefly, bioinformatics analysis was performed with an in-house pipeline (coreBM-Germline_1.0.0) following GATK best practices (13). Read alignment was conducted against the hg38 reference genome. Annotation of the variant calling files was carried out using the Jnomics platform (jnomics.es). Data analysis was based on custom panels testing for neurodevelopmental disorders (gene list available upon request). Variant interpretation and classification were performed according to the ACMG recommendations (14).

### 2.3 Statistical analysis

Descriptive statistics were obtained from the total sample and from the subgroups of participants (patients with findings in ES and patients with no findings) and were calculated using percentages, means, and standard deviations. Statistical tests were used to explore the possible effect of gender, IQ or age in the results. Fisher’s exact test was used to determine whether there was a significant association between two categorical variables when the sample size was small. All statistical analyses were performed using IBM SPSS Statistics for Windows, Version 18.0 (IBM, Chicago, IL, USA). The normality of continuous variables was tested using the Kolmogorov–Smirnov and Shapiro–Wilk tests, while the equality of the variance between the groups was assessed using Levene’s test. Two-tailed p-values < 0.05 were of statistical significance.

## 3 Results

### 3.1 Study cohort

In the present study, we reviewed the charts of 20 subjects diagnosed with ASD with a mean age of 11.95 years (SD= ± 2.7); 75% of the sample was male and 25% female. The mean age of ASD diagnosis was 4.65 years (SD= ± 2.8). According to the high clinical heterogeneity of ASD, 80% of our patients presented ID with a mean intellectual quotient (IQ) of 54.30 (SD= ± 22.2). Other co-occurring clinical conditions included ADHD, speech disorders, psychomotor delay, the presence of dysmorphic features and medical co-morbidities. The clinical description of the sample is reported in **Table 1**.

**Table 1:** Clinical description of the sample.

**Medical co-morbidities**	N=19 (95%)
**Intellectual disability**	N=16 (80%)
**Speech delay**	N=15 (75%)
**ADHD**	N=13 (65%)
**Dysmorphic features**	N=13 (65%)
**Psychomotor delay**	N=12 (60%)
**Speech disorders**	N=9 (45%)
**Enuresis**	N=9 (45%)
**Encopresis**	N=6 (30%)

N = number of patients. Percentage of sample were displayed in brackets.

### 3.2 Differences found between patients with findings in ES and patients with no findings

ES was performed on 20 patients. As shown in **Table 2**, a pathogenic variant was identified in 10 patients (50% of the sample collected). In 90% the pathomechanism was *de novo* variation. Patient 6 was found to carry an inherited pathogenic variant in the *ALX4* from his affected mother (**Table 3**).

**Table 2:** Clinical features of two groups.

	Pathogenic ES finding N=10	VUS or not significant finding N=10	Fisher exact test
**Dysmorphic traits**	(80%)	(50%)	0.350
**Dysmorphic eyes**	(50%)	(20%)	0.350
**Dysmorphic ears**	(30%)	(10%)	1.000
**Dysmorphic trunk**	(60%)	(0%)	**0.011**
**Dysmorphic hands/arms**	(40%)	(10%)	1.000
**Dysmorphic legs/feet**	(30%)	(0%)	0.211
**More than 3 dysmorphic features**	(60%)	(0%)	**0.011**
**Macrocephaly**	(20%)	(30%)	1.000
**Speech delay**	(60%)	(90%)	0.303
**Speech disorders**	(40%)	(50%)	1.000
**Psychomotor delay**	(70%)	(40%)	0.370
**Hypotonia**	(90%)	(10%)	**0.001**
**Hypotonia + psychomotor and wandering delay**	(80%)	(10%)	**0.005**
**Enuresis**	(30%)	(30%)	1.000
**Encopresis**	(50%)	(40%)	1.000
**Refractive defects and strabismus**	(80%)	(20%)	**0.023**
**Sleep disturbances**	70%)	(60%)	1.000
**Epilepsy**	(30%)	(10%)	0.582
**Obesity**	(10%)	(10%)	1.000
**Gastrointestinal disturbances**	(40%)	(60%)	0.656
**Heart diseases**	(10%)	(0%)	1.000
**Skin spots**	(20%)	(20%)	1.000
**Allergy**	(20%)	(40%)	0.628
**Psychiatric co-morbidities**	(80%)	(70%)	1.000
**Behavioral disturbances**	(50%)	(90%)	0.141
**ADHD**	(80%)	(60%)	1.000

Significant p values are highlighted in bold.

**Table 3:** Clinical features and genetic results of the diagnosed patients.

Case	Clinical findings	Gene (name; REFSEQ)	Variant	Known disease (OMIM)	Inheritance & origin
Patient 1	ID, hypotonia, dysmorphic features, speech and psychomotor delay, enuresis, refractive defects and ADHD, behavioral disturbances	*ADNP* (NM_181442.4)	c.2456dupA, p.(Tyr719Ter)	Helsmoortel-van der Aa syndrome (#615873)	AD *de novo*
Patient 2	Hypotonia, dysmorphic features, pointed palate, pectus excavatum, insomnia, dental decalcification, enuresis, strabismus, ADHD	*FBN1* (NM_000138.4)	c.5100delT, p.(Tyr1700Ter)	Weill-Marchesani syndrome 2 (#608328)	AD *de novo*
Patient 3	ID, hypotonia, macrocephaly, dysmorphic features, insomnia, ADHD, anxiety	*WAC* (NM_016628.4)	c.12801281delCTinsGAG, p.(Ser427Ter)	Desanto-Shinawi syndrome (#616708)	AD *de novo*
Patient 4	ID, hypotonia, microcephaly, distinctive craniofacial features, insomnia, severe feeding difficulties, failure to thrive, absent speech, ADHD, behavioral disturbances, strabismus	*ASXL3* (NM_030632.2)	c.1534_1535delTT, p.(lLeu512AlafsTer4)	Bainbrifge Ropers syndrome (#615485)	AD *de novo*
Patient 5	ID, hypotonia, psychomotor delay, absent speech, insomnia, epilepsy, spherocytosis, ADHD, behavioral disturbances	*NR4A2* (NM_006186.3)	c.327dup, p.(S110Vfs*2)	Intellectual developmental disorder with language impairment and early-onset DOPA-responsive dystonia-parkinsonism (#601828)	AD *de novo*
Patient 6	ID, hypotonia, macrocephaly, drooping eyelids, refractive defects, speech and motor delay, ADHD	*ALX4* (NM_021926.4)	c.17dup, p(Cys6TrpfsTer30)	Foramina parietal 2 (#609597)	AD Maternal inheritance
Patient 7	Hypotonia, refractive defects, recurrent otitis, short stature, triangular face, wide eyebrows, hypertelorism, prominent ears and nasal bridge, psychomotor delay, ADHD	*ANKRD11* (NM_013275.5)	c.226G>A, p.(Glu76Lys)	KBG syndrome (#148050)	AD *de novo*
Patient 8	ID, hypotonia, microcephaly, strabismus, distinctive craniofacial features, severe feeding difficulties, psychomotor delay	*POGZ* (NM_015100.4)	c.2989C>T, p.(Arg997Ter)	White-Sutton syndrome (#616364)	AD *de novo*
Patient 9	ID, hypotonia, strabismus, absent speech, insomnia, enuresis, psychomotor delay, epilepsy	*SHANK3* (NM_1372044.2)	c.3874G>T, p.(Glu1292Ter)	Phelan-McDermid syndrome (#606232)	AD *de novo*
Patient 10	Hypotonia, thymic hypoplasia, coarctation of the aorta, insomnia, psychomotor delay, ADHD, refractive defects	*BPTF* (NM_182641.27)	C.8562_8563del, p.(Val2855Serfs’Ter7)	Neurodevelopmental disorder with dysmorphic features and distal limb anomalies (#617755)	AD *de novo*

OMIM, Online Mendelian Inheritance in Man REFSEQ, the Reference Sequence database.

Four patients had no significant alteration and in 6 patients variants of unknown significance (VUS) were found. Statistical analyses did not identify significant differences between these two groups in gender, IQ or age. **Table 2** shows clinical features collected in both groups. Differences in co-occurring clinical conditions were analyzed. With respect to dysmorphic traits, there were no differences when we analyzed these globally, so they were analyzed separately. Patients with a positive finding in ES were more likely to present a dysmorphic trunk (p=0.011) and more than three dysmorphic features (p=0.011). Patients with findings in ES were more prone to present hypotonia (p=0.001) in the first months of life. No differences were found in relation to speech delay, speech disorders, psychomotor delay, enuresis, or encopresis. Patients with findings in ES were more likely to have hypotonia plus psychomotor and wandering delay (p=0.005). In relation to medical co-morbidities, patients with findings in ES were more likely to have refractive defects and strabismus (p=0.023). No differences were found between groups in relation to psychiatric co-morbidities or behavioral disturbances. **Table 3** summarizes the clinical features and genetic results of the diagnosed patients.

## 4 Discussion

In the present study, we performed ES in a cohort of 20 patients diagnosed with ASD and we detected pathogenic variants in 9 genes already known to be associated with ASD (ADNP, WAC, ASXL3, NR4A2, ALX4, ANKRD1, POGZ, SHANK3 and BPTF) and in 1 gene with not enough evidence *(FBN1).*

The *ADNP* gene has been found to be mutated in significant percentage of patients diagnosed with syndromic autism or intellectual disability and is one of its more frequent genetic causes. The biological role of ADNP is associated with essential functions such as organogenesis of the developing embryo and proper brain formation. Heterozygous and predicted loss-of-function ADNP mutations in individuals inevitably result in the clinical presentation with the Helsmoortel–Van der Aa syndrome and include intellectual disability of various severities, severely delayed speech including oral apraxia, behavioral problems, and motor development delays of various severities. Other ADNP symptoms may include delayed toilet training, pain insensitivity, sleep problems, seizures, structural brain anomalies, distinct dysmorphic facial features, sensory problems including mostly visual problems, occasional hearing problems, autistic traits and hypotonia or in rare cases, hypertonia. Gastrointestinal problems are most frequently encountered. Congenital heart defects, short stature, skin cell deficiencies and hormonal deficiencies/abnormalities as well as recurrent infections are also apparent. About 80% of the children harboring ADNP mutations show early deciduous dentation (15).

The FBN1 is the major constitutive element of extracellular microfibrils is fibrillin (encoded by *FBN1 gene).*Microfibrils containing fibrillin-1 provide long-term force-bearing structural support. The periphery of the elastic fiber is formed by microfibrils in tissues such as skin, blood vessels, and lung. Fibrillin-1 also has a critical role in homeostasis of tissue and regulates osteoblast maturation by controlling TGF-beta bioavailability and calibrating TGF-beta and bone morphogenetic protein levels. *FBN1* gene variants lead to different types of diseases including Weill-Marchesani syndrome 2. Weill-Marchesani syndrome (WMS) is a rare condition characterized by short stature, brachydactyly, joint stiffness, and characteristic eye abnormalities including microspherophakia, ectopia of the lens, severe myopia, and glaucoma (16).

The WW domain containing adaptor with coiled-coil (*WAC*) gene is associated with DeSanto–Shinawi syndrome (DESSH). The WAC protein can have many functions, including dimerizing, chromatin binding *and* RNA polymerase II complex binding. Those diagnosed with DESSH have symptoms of cranial dysmorphia and hypotonia with comorbidities including attention deficit hyperactivity disorder (ADHD), autism, and seizure susceptibility. Moreover, *WAC* variants rank it as a high-confidence autism spectrum disorder risk gene (17).

Chromatin Modifying Disorders (CMD) have emerged as one of the most rapidly expanding genetic disorders associated with ASD. It includes Bainbridge-Ropers Syndrome (BRS), caused by truncating variants in *ASXL3. ASXL* genes play a critical role in embryonic development and reading of posttranslational histone modifications. Phenotypically, patients presented hypotonia, delay in achieving independent sitting and walking, and marked language delay. Intellectual disability ranges from moderate to severe. Affected individuals may also display autistic features. Feeding difficulties requiring support are frequent. Distinct facial features include highly arched or delineated eyebrows and also synophrys, and frequently a highly arched palate. Patients may exhibited skeletal anomalies including scoliotic attitude, joint laxity, pectus excavatum or carinatum and ulnar deviation of wrists (18).

*NR4A2* gene encodes a member of the steroid-thyroid hormone-retinoid receptor superfamily. The encoded protein may act as a transcription factor. Mutations in this gene have been associated with disorders related to dopaminergic dysfunction such as Parkinson disease, schizophrenia, manic depression, and autism spectrum disorder. Patients could present delayed psychomotor development, varying levels (mild to severe) of intellectual disability (ID)/developmental delay (DD). Other features include epilepsy, speech/language impairment, behavioral problems, and movement disorder/hypotonia. Behavioral problems included autism, attention deficit–hyperactivity disorder, anxiety, and hyposensitivity (19).

Foramina parietal 2 is clinically characterized by intellectual disability, craniofacial anomalies and enlarged parietal foramina and autism. *ALX4* gene function is provides instructions for making a member of the homeobox protein family. Homeobox proteins direct the formation of body structures during early embryonic development. The ALX4 protein is necessary for normal development of the skull and formation of the head and face and also controls the activity of genes that regulate cell growth and division (proliferation), cell maturation and specialization (differentiation), cell movement (migration), and cell survival (20).

Haploinsufficiency of *ANKRD11* due to deletion or truncation mutations causes KBG syndrome, a rare genetic disorder characterized by intellectual disability, autism spectrum disorder, seizures and craniofacial abnormalities (triangular face, wide eyebrows with mild synophrys, hypertelorism, prominent ears and nasal bridge with bulbous nasal tip, long flat philtrum and thin upper lip). Other features include behavioral disturbances including hyperactivity and. Developmental delay includes delayed motor milestones and markedly delayed speech and is almost always present in all patients. *ANKRD11* encodes ankyrin repeat domain-containing protein 11. The extent of *ANKRD11* functions is yet to be determined, but it has been shown to be a crucial chromatin regulator that controls histone acetylation and gene expression during neural development (21).

*POGZ* gene mutations are thought to impair the ability of the POGZ protein to bind to chromatin. Presentation may be with neonatal hypotonia or developmental delay during infancy. The main features observed in these patients are developmental delay, intellectual disabilities, and neurobehavioral abnormalities (including autism spectrum disorder). Most individuals have been described with mild to moderate ID; but the spectrum ranges from low-normal intelligence to severe ID. Some mild cerebral malformations can be found. Common craniofacial characteristics include microcephaly, high and broad forehead, midface hypoplasia or retrusion, tented or triangular mouth with downturned corners of the mouth, a broad nasal root and flat nasal bridge. Additional features may include hypotonia, seizures, sleep apnea, sensorineural hearing impairment, visual defects (strabismus, refraction errors), tendency to be overweight, short stature and gastrointestinal difficulties (22).

Mutations in the *SHANK* family genes have been linked to syndromic and idiopathic autism spectrum disorder, as well as to other neuropsychiatric and neurodevelopmental disorders (schizophrenia and intellectual disability). *SHANK3* encodes a synaptic scaffolding protein. The severity of the developmental delay tends to vary with deletion size, with most individual functioning in the severe to profound range. Major milestones are delayed, receptive language skills typically exceed expressive skills, and many individuals are nonverbal. Other neurological features include seizures, ataxic gait, sleep disturbance, abnormal brain imaging, and regression or loss of skills. Common physical traits include long eye lashes, large or unusual ears, relatively large hands, dysplastic toenails, full brow, dolicocephaly, full cheeks, bulbous nose, and pointed chin (23).

BPTF is a protein that in humans is encoded by the *BPTF* gene. Are regulatory subunits in the NURF-1 and NURF-5 ISWI chromatin remodeling complexes that assemble ordered nucleosome arrays to access DNA during replication, transcription, and repair. Clinical features include dysmorphic faces and distal limb anomalies, defined primarily by developmental delay/intellectual disability, speech delay, postnatal microcephaly, and dysmorphic features (24).

In **Table 3** clinical features and genetic results found in our patients were described and correlated with the description of the known diseases found in literature (15–24).

Identifying the etiology of ASD is difficult due to the high genetic heterogeneity of this condition. ASD often coexists with intellectual disability, with 70% of ASD patients also suffering from ID, whereas 40% of ID patients have ASD (25). However, differentiating between these clinically overlapping conditions with huge genetic heterogeneity is very difficult and up to 50% of patients suffering from ID and/or ASD remain molecularly undiagnosed (26). In individuals with ASD, *de novo* mutations in ES have previously been shown to be significantly correlated with lower IQ, but studies with other comorbidities are scarce. Interestingly we found developmental or clinical differences between ASD patients with pathogenic findings in ES and those with normal findings.

Respects to variants of unknown significance (VUS), 6 patients presented a VUS. VUS are genetic variants which are genes are already associated with ASD, but the specific altered regions do not have sufficient clinical evidence or functional shreds of evidence in order to be categorized in a pathogenic or benign variant. In other situations, some VUS seems to be relevant for clinical phenotype, but they are located at genes with few associations with ASD (27). For future research, it would be important to collect VUS found and describe accurately the phenotype of the patients in order to correlate it with pathogenicity.

### 4.1 Dysmorphic traits

Individuals with ASD often have a number of unusual physical characteristics, called dysmorphologies or minor physical abnormalities (MPA), such as deviations in the morphology of the head, eyes, ears, mouth, hands, and feet. As the brain and skin are derived from the same neuroectodermal layer during early fetal development, MPAs may mirror altered brain development (28). Therefore, the presence of multiple MPAs could suggest the possibility of an underlying genetic and/or environmental perturbation affecting embryogenesis. Multiple MPAs have been found in approximately 20% of individuals with ASD (29). To date the main limitation of MPA studies in ASD is that these studies used physical examinations not originally designed for MPA assessment. In our study 65% of the sample had MPAs. When we analyzed these in patients with a pathogenic ES finding, we found significant differences in trunk dysmorphia and in the number of dysmorphic traits. As Ozgen et al. reported, the presence of any of three abnormal physical features can indicate a genetic cause of ASD (30).

### 4.2 Motor impairments

In our study we found that patients with hypotonia, psychomotor and wandering delay were more likely to have pathogenic variants in genes when ES was performed. Although ASD is not perceived as a syndrome with obvious motor impairment, various motor coordination and programming deficits have been observed. Many studies have reported motor deficits including alterations in motor milestone development, clumsiness, motor incoordination, disturbances in reach-to-grasp movement, deficits in gross and fine motor movement and hypotonia (31, 32). Hypotonia may be defined as reduced resistance to passive range of motion (aphasic tone), or loss of postural control. It occurs in multiple neuromuscular, metabolic and genetic disorders and can be a sign of global developmental delay that may pre-dispose to a cognitive disability (33). The prevalence of hypotonia in two cohorts of patients diagnosed with ASD ranged from 25 to 51% (34, 35). In our study the prevalence (50%) was consistent with previous studies. In patients with a genetic finding the prevalence increased to 90% of the sample. Previous studies showed that patients with ASD have higher diagnostic yields for ES than for CMA (36, 37). With respect to psychomotor and wandering delay, preliminary evidence suggests that motor difficulties may be observed in between 50% and 79% of individuals with ASD (38, 39). Fifty-five percent of our sample exhibited psychomotor delay and 45% exhibited hypotonia and psychomotor and wandering delay. In patients with a genetic finding the prevalence increased to 70% with psychomotor delay and 80% with hypotonia, psychomotor and wandering delay. A previous study concluded that diminished motor skills, like ID, also correlate significantly with *de novo* mutations in ASD, and are an even more sensitive indicator of the damage of a *de novo* mutation than is ID (40).

### 4.3 Refractive defects and strabismus

Children and adolescents with ASD have a high prevalence of co-occurring medical conditions and multiple psychiatric disorders. The prevalence of ophthalmologic disorders was found to be more frequent among these patients than in the general pediatric population. The most frequent eye disorders identified in ASD patients are strabismus, nystagmus, and refractive errors or amblyopia. We detected refractive defects or strabismus in 50% of patients, similar to the rate found in a previous study (41). In our study, patients with a pathological finding in ES had more refractive defects and strabismus. Like ASD, refractive defects and strabismus occur in part due to genetic factors. Many of these genes are involved in common biological pathways known to mediate extracellular matrix composition and regulate connective tissue remodeling. Other associated genomic regions suggest novel mechanisms in the etiology of human myopia, such as mitochondrial-mediated cell death or photoreceptor-mediated visual signal transmission. Taken together, observational and experimental studies have revealed the complex nature of human refractive variation, which likely involves variants in several genes and functional pathways. These factors are complex and likely involve interactions between multiple genes (42, 43).

## 5 Limitations

The main limitation of the study was the small sample size, because of the study design (patients with a previously abnormal karyotype in peripheral blood, a positive FXS study, or a confirmed suspicion of an identifiable syndromic entity were excluded from the study) and the strict criteria of our hospital to perform exome sequencing (only ten ASD’s patients per year). For future studies, a large sample size will be interesting. Another limitation might be diagnostic methods. It would be important to develop algorithms based on clinical features, polygenic risk scores and comorbid conditions associated with ASD in order to best request exome analysis.

## 6 Conclusions

ES offers expanded diagnostic options for patients with ASD who are negative on CMA. The selection of patients with other co-morbidities such as hypotonia, various dysmorphic features or refractive defects/strabismus might increase the diagnostic yield.

However, further studies are needed for a better understanding of the ASD etiology and also the different phenotypes. It will also be important to develop more accurate diagnostics that include hypotonia, refractive errors and strabismus as ES markers.

## Data Availability

The original contributions presented in the study are included in the article/supplementary material. Further inquiries can be directed to the corresponding author.
